# Epigenetic landscape underlying plant-microbiome chemical communication

**DOI:** 10.1093/ismejo/wraf249

**Published:** 2025-11-03

**Authors:** Fangze Gui, Yusufjon Gafforov, Juan Ignacio Vílchez, Jiangtao Zhao, Zhonghua Ma, Tianxing Lv, Mengcen Wang

**Affiliations:** College of Agriculture and Biotechnology, Zhejiang University, Hangzhou, Zhejiang 310058, China; State Key Laboratory of Rice Biology and Breeding, Ministry of Agricultural and Rural Affairs Laboratory of Molecular Biology of Crop Pathogens and Insects Pests, Zhejiang University, Hangzhou, Zhejiang 310058, China; Zhejiang Key Laboratory of Biology and Ecological Regulation of Crop Pathogens and Insects, Zhejiang Engineering Research Center for Biological Control of Crop Pathogens and Insect Pests, Institute of Pesticide and Environmental Toxicology, Zhejiang University, Hangzhou, Zhejiang 310058, China; Central Asian Center for Development Studies, New Uzbekistan University, Tashkent 100007, Uzbekistan; Faculty of Chemical Technology, Fergana State Technical University, Fergana, Fergana 150100, Uzbekistan; iPlantMicro Laboratory Oeiras, Instituto de Tecnologia Química e Biológica (ITQB)-NOVA, Oeiras, Lisboa 2780-157, Portugal; College of Agriculture and Biotechnology, Zhejiang University, Hangzhou, Zhejiang 310058, China; State Key Laboratory of Rice Biology and Breeding, Ministry of Agricultural and Rural Affairs Laboratory of Molecular Biology of Crop Pathogens and Insects Pests, Zhejiang University, Hangzhou, Zhejiang 310058, China; Zhejiang Key Laboratory of Biology and Ecological Regulation of Crop Pathogens and Insects, Zhejiang Engineering Research Center for Biological Control of Crop Pathogens and Insect Pests, Institute of Pesticide and Environmental Toxicology, Zhejiang University, Hangzhou, Zhejiang 310058, China; College of Agriculture and Biotechnology, Zhejiang University, Hangzhou, Zhejiang 310058, China; State Key Laboratory of Rice Biology and Breeding, Ministry of Agricultural and Rural Affairs Laboratory of Molecular Biology of Crop Pathogens and Insects Pests, Zhejiang University, Hangzhou, Zhejiang 310058, China; College of Agriculture and Biotechnology, Zhejiang University, Hangzhou, Zhejiang 310058, China; State Key Laboratory of Rice Biology and Breeding, Ministry of Agricultural and Rural Affairs Laboratory of Molecular Biology of Crop Pathogens and Insects Pests, Zhejiang University, Hangzhou, Zhejiang 310058, China; Zhejiang Key Laboratory of Biology and Ecological Regulation of Crop Pathogens and Insects, Zhejiang Engineering Research Center for Biological Control of Crop Pathogens and Insect Pests, Institute of Pesticide and Environmental Toxicology, Zhejiang University, Hangzhou, Zhejiang 310058, China; College of Agriculture and Biotechnology, Zhejiang University, Hangzhou, Zhejiang 310058, China; State Key Laboratory of Rice Biology and Breeding, Ministry of Agricultural and Rural Affairs Laboratory of Molecular Biology of Crop Pathogens and Insects Pests, Zhejiang University, Hangzhou, Zhejiang 310058, China; Zhejiang Key Laboratory of Biology and Ecological Regulation of Crop Pathogens and Insects, Zhejiang Engineering Research Center for Biological Control of Crop Pathogens and Insect Pests, Institute of Pesticide and Environmental Toxicology, Zhejiang University, Hangzhou, Zhejiang 310058, China; Global Education Program for AgriScience Frontiers, Graduate School of Agriculture, Hokkaido University, Sapporo, Hokkaido 060-8589, Japan

**Keywords:** plant-microbiome interactions, epigenetics, chemical communication, click chemistry, sustainable agriculture

## Abstract

Chemical communication, a universal mode among the interactive members within dynamic plant-microbiome systems, fundamentally drives coevolutionary trajectories. Emerging evidence suggests the critical role of epigenetic regulation in chemical communication, though its mechanistic insights are yet not well understood, a gap that has limited the precise mining of microbiomes function in modern agriculture. Here, we synthesize recent findings from chemistry to epigenetics to illuminate the overlooked epigenetic landscape in plant-microbiome chemical communication. Revisiting the traditional plant-pathogen interaction model and a more complex ternary model involving the plant resident microbiota, we not only present knowledge gaps but also critically dissect the paradoxical roles of resident microbiota by proposing four chemo-epigenetic patterns that fine-tune the interactions among plants, resident microbiota and pathogens. Further, Intelligent Click Chemistry, an innovative interdisciplinary strategy integrating click chemistry and artificial intelligence, is proposed and discussed, with the aim of unraveling the complex chemo-epigenetic events underlying plant-microbiome chemical communication. Untangling the epigenetic landscape underpinning plant-microbiome chemical communication would enable the strategic and precise exploitation of beneficial microbial traits and suppression of detrimental interactions for sustainable agriculture.

## Introduction

Plant-microbiome systems, serving as a pivotal nexus in carbon sequestration, nutrient cycling and ecosystem resilience, play an essential role in sustainable agriculture and food security [1]. Chemical communication, a universal mode among the interactive members within dynamic plant-microbiome systems [2], fundamentally drives coevolutionary trajectories, and by intertwining with epigenetic regulation, orchestrates adaptive responses in both plants and their associated microbiota. Despite the critical role of epigenetic regulation in chemical communication, its underlying mechanisms are yet to be fully elucidated [3, 4], which has limited effective mining of the plant-microbiome interactions in modern agriculture.

As a sophisticated process, chemical communication in plant-microbiome systems involves three key steps, including chemical molecule production, perception and response [5]. In this context, chemical molecules broadly include small molecules and biological macromolecules that participate in interspecies signaling. To detect pathogenic microbial invasion, plants perceive conserved chemical molecules known as pathogen-associated molecular patterns, such as flagellin proteins, peptidoglycan, and elongation factor-Tu through pattern recognition receptors (PRRs) [6]. Upon recognition, PRRs initiate a defense mechanism termed pathogen-triggered immunity (PTI), which serves as the plant’s first line of defense against microbial invasion. In turn, pathogenic microbes employ specialized secretion systems to directly deliver effectors into the host cell cytoplasm or fuse secretory vesicles with host cells [7]. Effectors are chemical weapons used by pathogens to suppress host defense, leading to effector-triggered susceptibility (ETS) [8]. To counteract this, plants have evolved a second layer of defense through resistance (*R*) proteins that specifically recognize these microbial effectors or their activity, triggering a stronger immune response termed effector-triggered immunity (ETI) that leads to hypersensitive response and systemic resistance [9, 10]. This dynamic co-evolutionary arms race between plants and pathogens, conceptualized as the Zig-Zag model [10], highlights the binary interplay between plant defense and pathogen virulence.

Emerging studies over the past decade suggest that plant-microbiome interactions extend beyond the classical binary framework to form a complex ternary network involving the resident microbiota [11–14]. The resident microbiota is widely recognized to play essential roles in host physiology, from regulating growth to bolstering disease resistance [15–18]. However, despite that emerging evidence also implies its functional duality-switching between a symbiotic ally and a pathogen accomplice during infection, especially under global change scenarios [19, 20]. A comprehensive understanding of epigenetic processes in such tripartite systems is essential for enabling precision engineering of disease-suppressive microbiomes, offering new avenues to secure food production beyond conventional genetic breeding.

**Figure 1:**
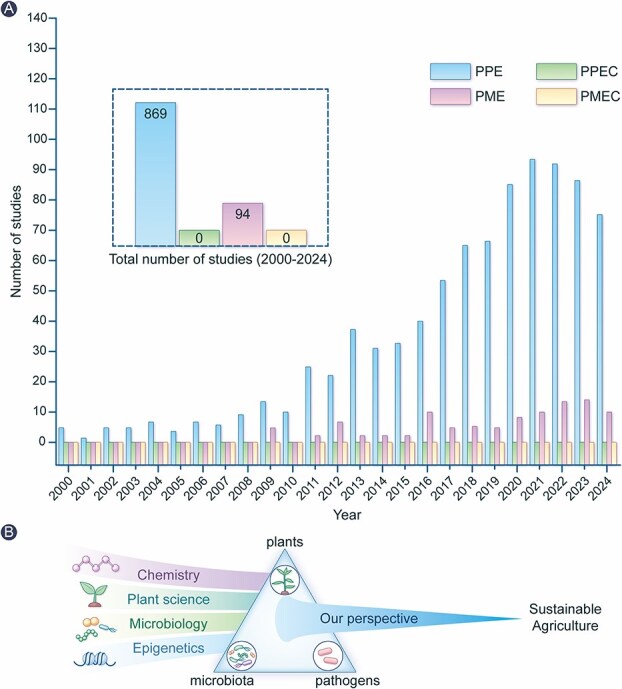
Interdisciplinary research landscape of plant-microbe interactions from 2000 to 2024. Publications were retrieved from the web of science database under the “plant sciences” category using the following topic queries (A): Plant pathogen epigenetic (PPE); plant pathogen epigenetic AND chemistry (PPEC); plant microbe epigenetic (PME); and plant microbiome epigenetic AND chemistry (PMEC). Interdisciplinary understanding of chemistry, plant science, microbiology and epigenetics is essential to enable the effective future engineering of plant-microbiome interactions for sustainable agriculture (B).

In the past decades, plant-microbiome interactions have remained largely underexplored from chemistry to epigenetics (Fig. 1A). Interdisciplinary understanding of chemistry, plant science, microbiology and epigenetics is essential to enable the effective future engineering of plant-microbiome interactions for sustainable agriculture (Fig. 1B). Here, we emphasize the epigenetic landscape hidden in plant-microbiome chemical communication. Revisiting the conventional plant-pathogen binary model, we identify key knowledge gaps and its broader links to climate change. Moving to a ternary framework, we explore the paradoxical roles of resident microbiota by proposing four chemo-epigenetic patterns that fine-tune the interactions among plants, resident microbiota and pathogens. To tackle these complexities, we also introduce Intelligent Click Chemistry (ICC), an interdisciplinary strategy integrating click chemistry with artificial intelligence (AI). This article offers new opportunities to understand the plant-microbiome interactions from chemistry to epigenetics, enabling the strategic harnessing of beneficial traits while mitigating detrimental ones toward sustainable agriculture.

### Epigenetic dimensions of pathogen-plant coevolution

**Figure 2:**
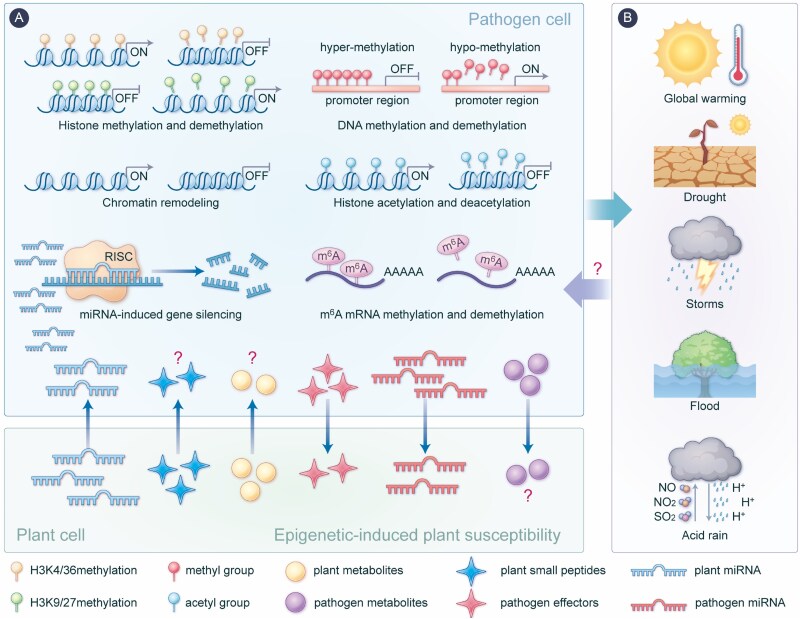
Epigenetic landscape in the chemical signaling between pathogens and plants. Fungal, bacterial, viral, and nematode pathogens, representing diverse plant pathogens, have evolved to employ epigenetic mechanisms to regulate virulence. During the process of pathogen-plant binary interactions, plant and microbial secreted bioactive molecules, such as plant peptides, pathogen effectors, plant and pathogen small molecule metabolites and miRNAs, may play a critical role in epigenetic regulation. These molecules influence pathogens pathogenicity and plant phenotypes, and contribute to shaping the evolutionary outcomes of pathogen-plant coevolution (A). Moreover, in the context of global climate change, epigenetic modifications could be essential for enabling pathogens to adjust their pathogenic strategies and enhance their survival under various environmental stressors. However, it is still not fully understood how pathogens adapt their fitness in the face of fluctuating environmental conditions through the epigenetic modification, ensuring their continued virulence amidst the challenges of a changing climate (B).

As sessile organisms, plants face relentless challenges from a diverse array of pathogens [3]. Over the course of coevolution, plants have developed a sophisticated two-layered immune system, PTI and ETI, to counteract pathogen attacks [10]. Conversely, pathogens have evolved various infection strategies, including ETS, to suppress or evade plant immune responses [21]. These dynamic, bidirectional interactions have positioned epigenetic regulation as a pivotal mechanism in shaping the outcomes of pathogen-plant coevolution [22–24]. By reprogramming gene expression and influencing adaptive responses, epigenetic processes provide profound insights into the chemical coevolutionary dynamics between these two biological systems (Fig. 2).

#### Pathogen pathogenicity regulation

There is increasing evidence that epigenetic regulation can precisely modulate the expression of pathogenic genes, thereby influencing the pathogenicity of phytopathogens in diverse crops [4, 25]. In gramineous crops, mycotoxin-producing pathogens have emerged as a major threat to global food security in recent years. In *Fusarium graminearum*, a key producer of the mycotoxin deoxynivalenol (DON), the histone acetyltransferase Gcn5, a core component of the Spt-Ada-Gcn5 acetyltransferase (SAGA) complex, is essential for both perithecia formation and DON biosynthesis by regulating the expression of specific target genes. Similarly, the histone acetyltransferase FgSAS3 is also indispensable for DON production and is required for successful host infection [26]. Moreover, another common type of histone modification, histone methylation is specifically enriched at loci associated with gibberellic acid (GA) biosynthesis in the fungal pathogen *Fusarium fujikuroi*, thereby promoting the expression of GA-related genes and enhancing GA production, which is critical for fungal virulence [27]. In addition, histone H3 lysine K4 (H3K4) methylation at the regulatory region of the conidiation-specific transcription factor Aba1 has been shown to stimulate fungal conidiation, underscoring the pivotal role of epigenetic marks in regulating key developmental and pathogenic processes [27]. Beyond H3K4 methylation, the activating mark H3K36 methylation also plays a crucial role in promoting the transcription of virulence-related genes in plant pathogenic fungi [28, 29]. In contrast, repressive histone modifications such as H3K27 methylation and H3K9 methylation generally silence gene expression through the establishment of heterochromatin, suggesting the site-specific regulatory patterns in histone methylation. These repressive modifications are also essential for the pathogenicity and infection processes of plant pathogenic fungi [30–32].

Epigenetic modifications have also been implicated in regulating virulence in pathogens of dicotyledonous crops, such as cotton. In *Verticillium dahliae*, a major fungal pathogen of cotton, DNA methylation at the 5′ position (5-mC) would repress the expression of *VdRim15* gene, which further promotes the full virulence of *V. dahliae* in host plants, and the attenuation of *V. dahliae* pathogenicity was also observed when treated with the DNA methylation inhibitor 5-Aza-2-deoxycytidine [33].

Besides histone modification and DNA methylation, other epigenetic modifications, such as chromatin remodeling [34, 35] and N^6^-methyladenosine (m^6^A) RNA modification [36] also impact pathogen pathogenicity (Fig. 2A). Despite a comprehensive understanding of the epigenetic modulation of virulence-associated genes, the persistence of pathogenicity in pathogens is increasingly influenced by their ability to adapt to the challenges posed by global climate change [37] Factors such as rising temperatures, prolonged droughts, and shifting humidity patterns are altering environmental conditions at an unprecedented rate (Fig. 2B). Epigenetic modifications could play a pivotal role in enabling pathogens to adjust their pathogenic strategies and enhance survival under these stressors. Future research, focusing on how pathogens exploit epigenetic mechanisms to rapidly respond to these external perturbations and regulate their pathogenicity, growth, and development, will shed light on understanding how pathogens adapt their fitness in the face of fluctuating environmental conditions through the epigenetic modification, and ensure their continued virulence amidst the challenges of a changing climate (Fig. 2B).

#### Plant defense versus pathogen counter-defense

Epigenetic regulation directly participates in plant immune responses via transcriptional reprograming of defense-response genes upon pathogen infection [4, 38, 39]. A recent study indicates that the m^6^A demethylase AhALKBH15 in host peanut can remove m^6^A modification of the disease-resistance gene *AhCQ2G6Y*, which subsequently upregulates this gene expression and promote host resistance to bacterial wilt caused by *Ralstonia solanacearum* [40]. Histone acetyltransferases Elongator subunit 2 (ELP2) and ELP3 have also been reported to positively regulate host immunity during pathogen attack through altering histone acetylation levels in the JA/ET defense pathway marker gene *PLANT DEFENSIN1.2* (*PDF1.2*) [41]. In addition, DNA and histone methylation and chromatin remodeling are also critically involved in regulating plant immunity [4, 42–44]. Apart from enhancing their immunity through self-regulation of epigenetic levels, plants also employ the secretion of small RNA such as microRNAs (miRNAs) for interspecies communication [45–47], thereby mitigating the pathogenicity of pathogens as part of their defense strategy. However, whether/how the plant-secreted chemical molecules, such as small peptides and secondary metabolites, epigenetically regulate pathogen growth and virulence remains not well-understood (Fig. 2A).

Similar to fungal pathogens, bacterial [48], viral [49, 50], and nematode pathogens [51] have also evolved to employ epigenetic mechanisms to regulate virulence, reflecting a conserved strategy across diverse evolutionary lineages [38]. Viruses, which lack autonomous chromatin systems, often exploit host-based epigenetic machinery to facilitate infection [52]. Therefore, these distinctions underscore that while the core epigenetic mechanisms are evolutionarily conserved, their utilization and biological contexts have diversified across pathogen groups (Fig. 2A).

Beyond their role in modulating plant defense-related genes that mediate plant resistance, epigenetic components may also contribute to host plant susceptibility to pathogens by negatively regulating the plant immune system, thereby enhancing pathogen survival and dispersal [53, 54]. Pathogens continuously coevolve with plants, developing sophisticated epigenetic strategies to counter defense by manipulating host susceptibility. Pathogens secrete effector proteins to manipulate host epigenetic processes, inducing ETS. The effector UvSec117 secreted by pathogen *Ustilaginoidea virens* can target the rice histone deacetylase OsHDA701, leading to the reduced histone H3K9 acetylation levels in rice and suppressed defense gene activation [55]. Moreover, the Foc-milR138, induced during infection by *Fusarium oxysporum* f. sp. *cubense* (Foc), silences the receptor-like kinase MaLYK3 in the host, suppressing immunity and promoting infection [56]. These examples highlight that most epigenetic manipulations are mediated by macromolecular effectors or *trans-*kingdom small RNAs, typically dependent on direct pathogen-host cell contact. However, pathogens are also known to secrete diffusible small molecule metabolites (e.g. mycotoxins) during infection. While these chemical molecules are capable of translocating into plant cells through passive diffusion, active transportation and endocytosis [57], influencing defense and nutrient acquisition pathways without direct contact, their potential role in epigenetic regulation remains an enigmatic aspect of plant-pathogen interactions, warranting further exploration (Fig. 2A).

### Resident microbiota interconnects plant-pathogen interactions

**Figure 3:**
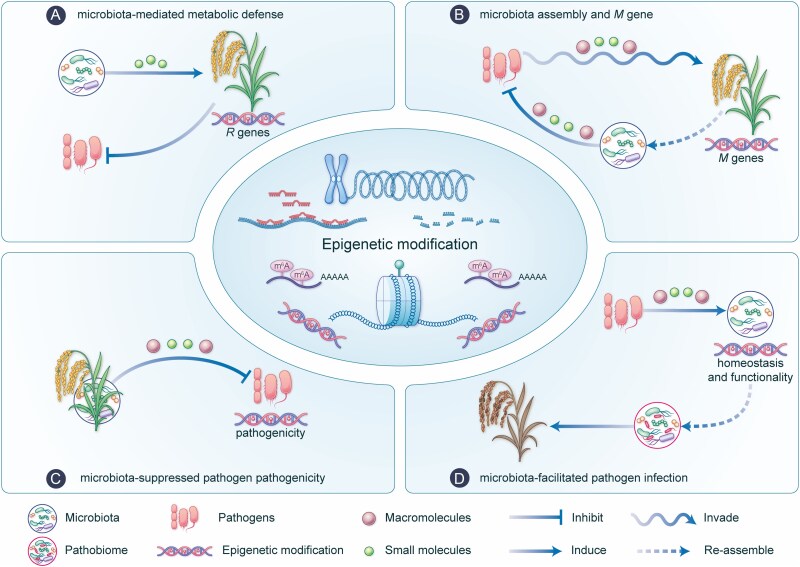
The proposed chemo-epigenetic patterns underlying multivariate plant-microbiome interactions. Epigenetic modification underlying multivariate plant-microbiome chemical signaling are pivotal in shaping the dynamics and outcome of pathogen infection through four patterns, including microbiota-mediated metabolic defense (A), microbiota assembly and *M* gene (B), microbiota-suppressed pathogen pathogenicity (C), and microbiota-facilitated pathogen infection (D). The proposed epigenetic modification patterns behind the disease resistance and susceptibility are mediated by complex chemical signaling molecules derived from microbes.

Plant phenotypes, including resistance and susceptibility to pathogens, are shaped by the co-regulated expression of genes from both the host plant and its resident microbiota [58–60]. This intricate interplay highlights the resident microbiota’s role in modulating gene expression, which can re-shape plant-pathogen interactions [61–63]. Through the complex chemical molecules, the ternary interactions among plants, their resident microbiota, and pathogens was dynamically modulated via epigenetic drivers. However, the chemical language-governed epigenetic modifications underpinning the ternary systems are yet poorly understood. Here, we propose a conceptual framework (Fig. 3) to explore the overlooked epigenetic drivers that exists within four patterns of the resident microbiota-associated interactive dynamics, with the potential to refine crop improvement-targeted strategies by enhancing resistance or reducing susceptibility.

#### Microbiota-mediated metabolic defense

Plant microbiota is known to act as an extension of the plant immune system with the capacity to reshape the plant-pathogen interactions [64, 65]. A ternary interaction model mediated by the plant-microbiome indicates that the consortium of *Chitinophaga* and *Flavobacterium*, enriched in the sugar beet that grows in disease-suppressive soils, could consistently suppress the wilt fungus *Rhizoctonia solani* through antifungal enzymes and secondary metabolites [66]. The microbiota-mediated metabolic defense was also observed in rice. Unlike the direct antifungal metabolic defense in sugar beet, the resident microbiota reprograms the innate metabolism of branched-chain amino acids (BCAA) in rice panicle to interfere with the pathogen invasion. Mechanistic analyses reveal that keystone microbial taxa enriched in the panicles of disease-suppressive plants, including *Lactobacillus* spp. and *Aspergillus* spp., can suppress the expression of the branched-chain amino acid aminotransferase gene (*OsBCAT*), thereby elevating BCAA levels in rice panicles. This metabolic shift ultimately confers disease resistance by triggering apoptosis-like cell death in *U. virens* [67]. This observation suggests the metabolic defense is closely linked to the altered expression of *OsBCAT*, but the chemical molecules derived from the beneficial microbes that regulate this process is uncharacterized, as well as whether it is governed at the transcriptional or epigenetic level, remain unanswered [68].

While we recognize that resident microbiota modulates the expression of pathogen-defense genes (e.g. *R* genes) in their hosts (Fig. 3A), the mechanisms underlying plant-resident microbiota-pathogen interactions are only beginning to be uncovered. To determine whether epigenetic modifications function as a broad-spectrum strategy against pathogen infection, it is crucial to identify the host-protective metabolites produced by the resident microbiota and evaluate their potential to trigger epigenetic modifications in defense-related genes of host plants (Fig. 3A).

#### Microbiota assembly and *M* gene

The plants also assemble the disease-suppressive microbial communities in response to pathogen infection [69–73], in association with the epigenetic modification observed in the hosts. In *Arabidopsis*, the Dicer-like proteins play an important role in assembling root microbiota by regulating the biogenesis of small RNAs [74]. As a symbol of the impaired root microbiota assembly, the decreased amplicon sequence variants (ASVs) of root microbiota occurs in *ibm1* (INCREASE IN BONSAI METHYLATION 1) mutants, despite that dysfunction of plant histone demethylase (IBM1) caused salicylic acid–mediated activation of autoimmunity, the accumulation of defensive metabolites as well as the enhanced resistance to *Pseudomonas syringae* DC3000 [75]. Such DNA methylation can be antagonized by the active DNA demethylation through controlling *myo*-inositol homeostasis, which is also capable of restoring the mutualism between the beneficial root bacteria and tomato [76]. While DNA methylation has been shown to negatively impact microbiota assembly, these observations are primarily based on dicotyledonous plants, such as *Arabidopsis* and tomato. The roles of other types of epigenetic modifications, as well as their effects on microbiota assembly in important crops like rice and wheat, remain largely unexplored.

Notably, microbiome-shaping genes (*M* genes), which are widely distributed and conserved across various plants, enable hosts to actively assemble a desirable microbiota alongside their innate immunity in response to pathogen infection [73, 77]. In rice, the haplotypes of *OsPAL02* (a phenylalanine/tyrosine ammonia-lyase gene) differ between *indica* and *japonica* rice and have been shown to regulate phyllosphere microbiota homeostasis and bacterial pathogen resistance. Both *OsPAL02* overexpression and knockout rice lines exhibit significant differences in microbiota assembly [71]. These observations suggest that *OsPAL02*, as a representative *M* gene, plays a dual role by not only participating in defense-related metabolic pathways but also modulating the organization of the plant-associated microbiota through its expression dynamics. Such findings imply that *M* genes can influence microbiota structuring beyond their genetic variation, possibly via pathogen-induced epigenetic regulation of gene expression (Fig. 3B). Pathogen-derived molecules, such as effectors and secondary metabolites with diverse chemical structures, which are secreted to facilitate infection, are likely in turn perceived by the plants as specific exogenous signals to epigenetically control *M* genes activation, thereby coordinating assembly of disease-suppressive microbiota to defend against the infection (Fig. 3B).

#### Microbiota-suppressed pathogen pathogenicity

In nature, the plant microbiota not only forms a close association with the host plant to combat pathogen challenges but also directly interacts with pathogens through functional molecules that attenuate their pathogenicity [78]. Within the wheat head microbiota, the resident bacterium *Pseudomonas piscium* produces the signaling metabolite phenazine-1-carboxamide (PCN), which diffuses into *F. graminearum* cells and targets the histone acetyltransferase FgGcn5 in the SAGA complex. By inhibiting FgGcn5’s acetyltransferase activity, PCN disrupts histone acetylation, represses gene expression, and ultimately suppresses fungal growth and pathogenicity [79]. Similarly, *Streptomyces*, another wheat-associated microbe, secretes the signaling molecule rapamycin, which inactivates the target of rapamycin (TOR) pathway in *F. graminearum*. This inactivation promotes 26S proteasome-mediated degradation of the fungal histone acetyltransferase Gcn5, a key negative regulator of autophagy. Since Gcn5 acetylates and suppresses the autophagy-related protein Atg8, its degradation triggers extensive fungal autophagy, further weakening fungal pathogenicity [80]. In rice, beneficial *Aspergillus* species from the leaf microbiota suppress *R. solani* infection by secreting the signaling molecule 2,4-di-tert-butylphenol (DTBP). DTBP effectively neutralizes ROS-dependent pathogenicity by deactivating bZIP-activated *AMT1* transcription in *R. solani* [81] although the precise ROS-driven epigenetic regulatory mechanisms remain unknown [82]. Functioning synergistically with small signaling molecules, RNA equips the plant microbiota with an additional layer of offense against fungal pathogens [83, 84]. An RNA interference (RNAi)-engineered strain of the beneficial fungus *Trichoderma harzianum* acts as an *sRNA/dsRNA* donor, capable of triggering cross-species gene silencing in RNAi-sensitive fungal pathogens, such as *Verticillium dahliae* and *Fusarium oxysporum*, thereby inhibiting their pathogenicity in crops [85].

These findings highlight that chemical communication is a major mode of interaction between the plant microbiota and invasive pathogens. Notably, the plant microbiota plays a crucial role in modulating the pathogenicity of invasive pathogens through epigenetic modifications (Fig. 3C). However, the mechanisms underlying the microbial functional molecules with epigenetic effects remain to be elucidated. A key question for future research is whether their molecular targets within the pathogen’s epigenetic landscape are pathogen species-specific or broadly conserved (Fig. 3C). The epigenetically resolved mechanism of plant microbiota-induced pathogenicity attenuation will have significant implications for the targeted engineering of resident microbiota-related functional chemical traits. Furthermore, it will accelerate the discovery of next-generation synbiotics that exert potent pathogenicity-neutralizing activity instead of direct microbicidal effects.

#### Microbiota-facilitated pathogen infection

As the intimate relationship between plants and their microbiota becomes widely recognized, a fascinating yet largely unexplored question arises: Have pathogens co-evolved to counteract or even subvert this joint defense between plant and microbiota? In recent years, the conventional “one pathogen–one disease” theory has been increasingly challenged with the growing understanding of the plant microbiome [86]. Notably, during pathogen invasion, shifts in microbial community composition are frequently observed. However, whether these changes result from an active strategy employed by the pathogen or a passive adaptation by the microbiota remains a subject of debate, particularly regarding their ecological significance [87]. Despite these uncertainties, the plant microbiota has been acknowledged as a game changer in shaping the outcome of pathogen infections in host plants, and emerging evidence suggests that resident microbiota members can also shift to a pathogenic state, form an alliance with invasive pathogens, to exacerbate disease occurrence and severity [87, 88].

Virulence is not solely an intrinsic trait of pathogens but can be dynamically reshaped through their cooperative interactions with microbial communities. In *P. syringae*, type III effectors, typically deployed by individual strains, collectively restored pathogenicity when distributed among non-virulent strains. Even more strikingly, transferring these effectors into *Pseudomonas fluorescens*, a normally beneficial bacterium, converted it into a pathogen [89]. A similar phenomenon has been observed in tomato and cotton, where the fungal pathogen *Verticillium dahliae* releases the virulence effector VdAve1, potentially enhancing the adaptability of opportunistic pathogens such as *Pantoea corrugata*, *Ralstonia* sp., and *Serratia* sp. This interaction fosters the formation of a pathobiome, facilitating infection and disease progression [90]. These findings reveal the potential of resident microbiota in cooperation with pathogens to promote the infection process, blurring the line between beneficial microbes and pathogens, as well as raising deeper questions about the regulatory mechanisms behind the state transitions of microbiota prior or post specific pathogens. Given that microbial effectors can interfere with host epigenetic machinery, an intriguing and largely unexplored question also arises (Fig. 3D): Do pathogens deploy interspecies or even interkingdom chemical signaling to maintain pathobiome homeostasis and functionality through epigenetic regulation?

### Intelligent click chemistry for harnessing chemo-epigenetics

Insight into the chemical essential behind the epigenetic landscape in plant-microbiome interactions offers a promising solution to sustainable agriculture, but is yet limited by the effective integration and utilization of interdisciplinary approaches. To address this issue, we propose an innovative strategy designated ICC by integrating AI with click chemistry.

**Figure 4:**
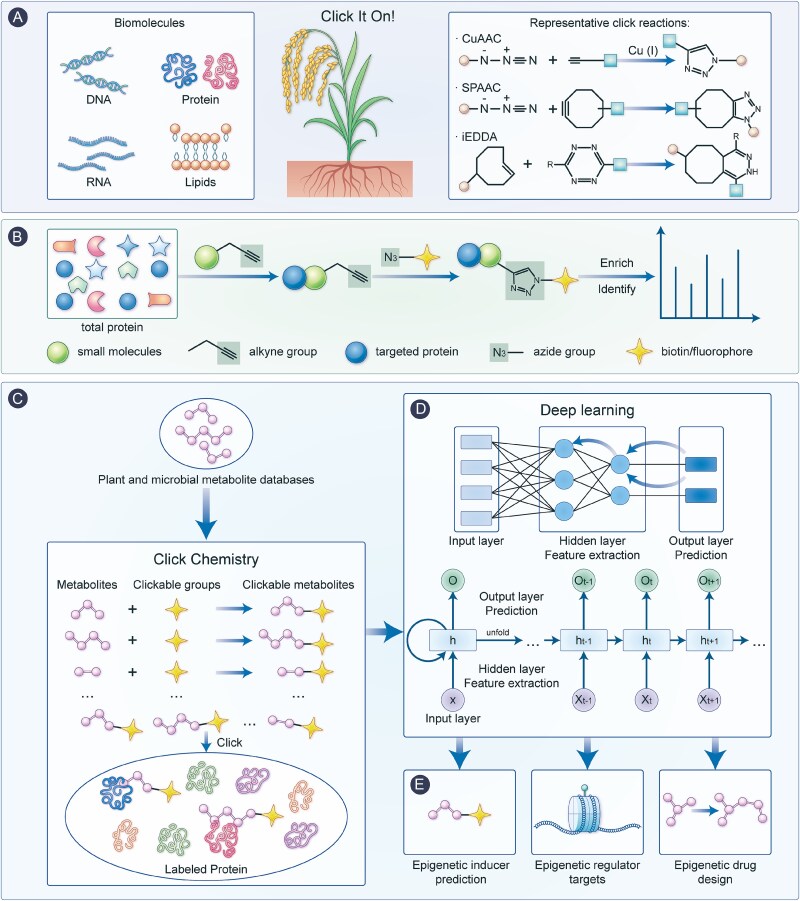
The process pipeline for the holistic ICC framework. Representative click reactions, such as CuAAC, SPAAC, iEDDA reactions, have been widely utilized in detection of biomolecules including DNA, RNA, protein and lipids (A). Moreover, in the research of plant proteome, click chemistry stands out as a robust approach. It plays a crucial role in addressing the key issues regarding the interaction between small molecules and protein by chemically modifying metabolites with alkyne groups and conjugating them with biotin or fluorophores, the protein targeted by small molecules can be effectively labeled. This tagging step facilitates further enrichment and identification of the protein (B). Chemical small molecules sourced from plant and microbial metabolite databases can be retrieved and feasibly labelled as probes to obtain their epigenetic regulators targets, thereby enabling the construction of complex interactions datasets between metabolites structures and the relevant epigenetic targets (C). Utilizing optimized neural network architectures, DL models extract and interpret large-scale datasets generated from click chemistry, and can be trained to predict whether the uncharacterized metabolites possess epigenetic activity. The input corresponds to the chemical structural information of the metabolites, the hidden layers extract relevant features such as molecular fingerprints, functional groups, topological descriptors, and the output yields the probability that the metabolite exhibits epigenetic-modulating potential (D). ICC aims to enable prediction of small molecules with epigenetic-inducing potential and the epigenetic regulators targeted by small molecules, and design of novel epigenetic drugs (E).

Click chemistry is a class of chemical reactions that facilitate the rapid and reliable formation of covalent bonds between molecules [91]. Representative click reactions, such as copper-catalyzed azide-alkyne cycloaddition (CuAAC), strain-promoted azide-alkyne cycloaddition (SPAAC), and inverse electron-demand Diels–Alder (iEDDA) reactions (Fig. 4A), have been widely utilized for in vitro detection of biomolecules such as DNA, RNA, protein and lipids [92]. Serving as a promising approach in the research of plant proteome, click chemistry offer opportunities to solve the key problems of the interaction between small molecules and proteins by using chemical probes to identify and profile protein [93]. By chemically modifying metabolites with clickable functional groups (e.g. azides or alkynes) and conjugating reporter tags (such as biotin or fluorophores), small molecule-targeting protein can be efficiently labeled, allowing for further enrichment and identification (Fig. 4B). Hence, click chemistry would be also applicable for advancing our understanding of plant and microbial secondary metabolites and the epigenetic effects that these metabolites may induce, but its application in investigating the complex and dynamic epigenetic effects within the context of multivariate plant-microbiome interactions is yet overlooked. However, it is remarkable that the interpretation of the pool of candidate epigenetic regulators, in particular, precise identification of genuine targets might be challenging, requiring time-consuming validation through extensive genetic approaches (e.g. knockout/knockdown experiments) and *in vivo/in vitro* biochemical assays to confirm direct interactions.

As a powerful tool for the computational analysis of large datasets [94], AI, particularly deep learning (DL), has recently been applied in the field of biology [95–97]. Integration of AI techniques would enable more efficient interpretation and utilization of click chemistry-derived datasets. In the ICC, the small molecules from plant and microbial metabolite databases, such as PCMD [98], PMHub [99] and the Natural Products Atlas [100], are retrieved and feasibly labelled as probes to obtain their epigenetic regulators targets, thereby enabling the construction of complex interactions datasets between metabolites structures and the relevant epigenetic targets [101] (Fig. 4C). Subsequently, by leveraging neural network architectures, DL models can autonomously analyze the abovementioned large-scale datasets generated via click chemistry, enabling the identification, extraction of key features, and classification of chemical profile [102]. More specifically, a DL model can be trained to predict whether the uncharacterized metabolites possess epigenetic activity [103]. The input corresponds to the chemical structural information of the metabolites, the hidden layers extract relevant features such as molecular fingerprints, functional groups, topological descriptors, and the output yields the probability that the metabolite exhibits epigenetic-modulating potential (Fig. 4D).

This approach not only facilitates the prediction of epigenetic activity in previously uncharacterized small molecules, but also aids in uncovering regulatory interactions between metabolites and their associated epigenetic targets. Based on the integration of click chemistry and artificial intelligence, the ICC can be employed to predict the metabolites with epigenetic-inducing potential, and to further explore the underlying epigenetic regulatory mechanisms between metabolites and metabolites-targeting epigenetic regulators. Profoundly, the ICC also facilitates the rational design and incorporation of specific functional groups or structural motifs capable of inducing or inhibiting epigenetic modifications, thus supporting the development of novel epigenetic drugs with precise regulatory effects (Fig. 4E).

### Concluding remarks and outlook

Global climate change has been predicted to continue with more drastic trends, posing a dire challenge to sustainable agriculture and planetary health. As the largest biological interface on Earth, plant-microbiome systems directly and indirectly influence nutrient dynamics and biogeochemical cycles, contributing to the stability of the biosphere. Although we are still at the inception of understanding the epigenetic landscape underlying the plant-microbiome chemical communication, future research will undoubtedly unravel these chemo-epigenetic patterns along with their mechanistic insights. Employing strategies such as ICC, which combine AI-driven predictive capabilities with the synthetic versatility of click chemistry, is expected to accelerate the discovery of chemical signaling molecules as well as the epigenetic elements responsible for their production, perception and response. This article not only promises to deepen our understanding of the epigenetic essentials in plant–microbiome chemical communication but also offers microbiome-based precision solutions aiming to enhance crop resilience in the face of global climate challenges.

## Data Availability

Data sharing not applicable to this article as no datasets were generated or analyzed during the current study.
